# The complex dynamics of therapeutic leave in Psychiatry

**DOI:** 10.1192/j.eurpsy.2024.423

**Published:** 2024-08-27

**Authors:** Â. S. Pinto, T. P. Soares, A. Marques

**Affiliations:** Department of Psychiatry, Vila Nova de Gaia/Espinho Hospital Center, Vila Nova de Gaia, Portugal

## Abstract

**Introduction:**

Therapeutic leave, the temporary and authorized absence of a patient from a psychiatric inpatient facility, is a practice rooted in the shift of mental illness towards more humane and recovery oriented care. This shift began to gain momentum in the mid-20th century, with the deinstitutionalization movement, which sought to treat psychiatric patients in less restrictive environments and facilitate their integration into the community. Today, therapeutic leave remains relevant in general psychiatry inpatient treatment facilities, and on an international level. It’s additionally used as a way to assess the progress and the stability of the patient outside the controlled environment that is the hospital, and to provide a gradual transition back into independent living and potential stressors of the outside world. But it is administered more on tradition and perceived benefits than on solid scientific grounding, reflecting a practice guided by clinical experience rather than empirical data or guidelines.

**Objectives:**

Our review aims to evaluate the existing body of research on therapeutic leave in general psychiatry inpatient units. We intend to identify the outcomes that have been studied, and assess the extent and nature of their impact.

**Methods:**

This scoping review was conducted through a comprehensive search of academic databases, including Google Scholar, PubMed, Embase, Cochrane Library, and PsycINFO. Search terms were carefully selected to capture relevant publications, and the results were screened for their pertinence to the review’s aims. Studies focused on forensic settings were excluded.

**Results:**

The literature on therapeutic leave is notably limited, and the prevalence of its utilization in clinical practice remains unclear. Scientific publications primarily address readmission rates, with two indicating an increased risk in patients granted leave during their inpatient treatment. However, one report suggested a potential reduction. Length of stay (LOS) was negatively impacted, with prolonged hospitalization in these patients shown in one report. Post-discharge emergency room visits seem unaffected. A rise in readmission rates and LOS typically suggests higher subsequent healthcare costs. However, findings from another study contradict this expectation, with reduction of costs post-initial inpatient treatment. The literature also explores the hazards linked to therapeutic leave, highlighting that a significant portion, between 30 to 80%, of inpatient suicides transpire during such leave. Additional concerns extend to non-fatal self-harm, as well as the possibility of patients causing harm to others or to property.

**Conclusions:**

Our review reveals a significant research gap in therapeutic leave’s effects, with a reduced number of outcomes studied and inconclusive findings. Future studies should aim to clarify these outcomes and eventually define therapeutic leave protocols.

**Disclosure of Interest:**

None Declared

